# The common and specific osteoarthritis gait characteristics: A quantitative grading system for OA associated gait changes in mice

**DOI:** 10.1016/j.ocarto.2026.100757

**Published:** 2026-02-18

**Authors:** Yajun Liu, Ashley Knebel, Jing Ding, Gregory Jay, Qian Chen

**Affiliations:** a Laboratory of Molecular Biology and Nanomedicine, Department of Orthopaedics, Warren Alpert Medical School of Brown University, Rhode Island Hospital, Brown University Health, Providence, RI 02912 USA; b Department of Emergency Medicine, Warren Alpert Medical School of Brown University, Rhode Island Hospital, Brown University Health, Providence, RI 02912, USA

**Keywords:** Osteoarthritis (OA), Aging-associated OA, Post-traumatic OA (PTOA), Mouse gait, Gait grading system

## Abstract

**Objective:**

Osteoarthritis (OA) severity in animal models has been quantified primarily through grading systems reflecting the changes of joint morphology. There is a lack of a uniform grading system to quantify the changes in motion. Here, we develop a quantitative, easy-to-use grading system for OA-associated gait changes in mice.

**Design:**

We characterized the mouse gait changes in a surgery induced post-traumatic OA (PTOA) model and aging-associated OA (aging-OA) models using DigiGait. This treadmill system captured running motions for video analysis. We developed and validated the OA Gait grading system with a quantitative formula indicating the extent of OA-related movement changes.

**Results:**

We found gait changes in both aging-OA and PTOA mouse models, consistent with OA cartilage degeneration and increasing OARSI (Osteoarthritis Research Society International) scores. The female aging-OA and male PTOA mice share common gait alterations including a significant decrease in stride time and stride length and a significant increase in stride frequency. These three gait changes compose the “Common OA Gait” in mice. In addition, aging-OA alters gait symmetry between forelimbs and hindlimbs while PTOA alters ataxia coefficient between left and right limbs. These “Specific OA Gait” may indicate the involvement of aging and/or injury in OA pathogenesis.

**Conclusions:**

The OA Gait grading system correlated with OA pathogenesis. In some instances of aging-OA, it preceded the histopathological changes indicated by the OARSI grading system. Thus, the OA Gait grading system may be widely used for detecting alteration of functional movement outcomes in a sensitive, quantitative, and mechanistic manner.

## 1 Introduction

Osteoarthritis (OA) is a joint degenerative disease with prominent phenotypes including articular cartilage degradation, chronic inflammation, and bone remodeling [[1], [2], [3]]. Knee joint OA causes pain and affects mobility as its primary outcome in patients [4,5]. The progression of OA has a strong correlation with gait mechanics, especially affecting the spatial-temporal parameters involved in motion [[6], [7], [8]]. The development of OA can result from either chronic inflammation or acute injury [9,10], usually defined as aging-associated OA (aging-OA) or post-traumatic OA (PTOA). Both types of OA can cause destruction of joint cartilage and pain, thus leading to different degrees of disability with significant impact on daily life.

In OA research, various mouse models have been developed to mimic OA phenotypes, such as surgical PTOA models [11] and transgenic aging-OA models [[12], [13], [14], [15]]. The extent of OA pathogenesis in mice has been quantified primarily through grading systems reflecting the changes of joint morphology. Such histopathology grading systems have become a gold standard for comparing OA progression [16], but require sacrificing animals before the joint tissues can be harvested. Furthermore, there is a lack of a uniform grading system to quantify the changes of movement during OA pathogenesis in live animals. Clinical studies indicated that, in some OA cases, there is a lack of correlation between cartilage degradation and pain, which needs to be revealed by behavioral analysis in animal models [[17], [18], [19]]. Such disconnections between clinical parameters and OA markers in animal models could hamper translational research in developing OA disease modifying drugs.

Various gait analysis equipment has been developed to evaluate mouse gait. Two types of gait instruments are commercially available, treadmill (DigiGait, TreadScan) and voluntary movements (CatWalk, Tekscan). Treadmill enables a mouse to run at a set speed, whereas voluntary locomotion enables a mouse to move forward voluntarily [20,21]. Rodent gait systems allow for behavioral analysis of locomotion in similar methods as clinical gait systems. Although it is a powerful tool, how to interpret its large amount of output data can be complicated. The gait data generated automatically by these instruments includes temporal parameters, spatial parameters, frequency, coefficient of variation, and various derivatives. Multiple studies used DigiGait system to collect gait data, but the data presentations are different with only a few overlapping parameters [[22], [23], [24], [25]]. Even though there is an increasing appearance of gait data in publications, it remains a challenge to compare and cross reference the various aspects of gait parameters.

To overcome these challenges, we developed a quantitative, easy-to-use grading system for OA associated gait changes in mice with DigiGait. We identified three Common OA Gait (COG) parameters including stride time, stride length, and stride frequency. The COG represents consistent gait changes among different OA models. Thus, the OA Gait grading system allows for a quantitative evaluation combining multiple gait parameters and comparisons of gait abnormalities over time or across different settings. We found that OA gait changes may be detected before histopathological changes via the Osteoarthritis Research Society International (OARSI) grading system in some age associated-OA models. Thus, gait changes may represent a more sensitive indicator for detecting early stages of age associated-OA.

## 2 Methods

### 2.1 Animals

Three different OA mouse models were used in this study (Supp Fig. 1A). The use of animals is approved by Brown University Health Animal Welfare Committee (Institutional Animal Care and Use Committee, CMTT# 503424). All animal experiments were performed in accordance with institutional guidelines at Rhode Island Hospital. For the PTOA model, knee joint instability was induced by the destabilization of medial meniscus (DMM) surgery on the right hindlimb of male *Col2a1-CreER*^*T*^ mice (Strain: 006774, The Jackson Laboratory, MA, US). At 3 months of age, mice were anesthetized and surgically disconnected the anterior attachment of the medial meniscus to the tibial plateau (DMM+) [11,26]. A separate group of unoperated mice were used as the non-surgery group (DMM-). For the aging-OA models, cartilage-specific miR-365 overexpression and inducible transgenic mouse models were used, including both female and male mice. *MiR-365-flox* mice in C57BL/6 background were generated at Brown University Mouse Transgenic and Gene Targeting Facility as previously described [15]. Overexpression of the stress responsive miR-365 in the articular cartilage tissues was achieved by crossing *miR-365-flox* mice with *Col2a1-Cre* mice (Strain: 003554, The Jackson Laboratory, MA, US) [27]. The inducible miR-365 model was achieved by crossing *miR-365-flox* mice with *Col2a1-CreER*^*T*^ mice [28] and under tamoxifen (T5648, Sigma-Aldrich, MA, US) activation. More details are described in Supp Method.

### 2.2 Gait analysis and scoring

**Fig. 1:**
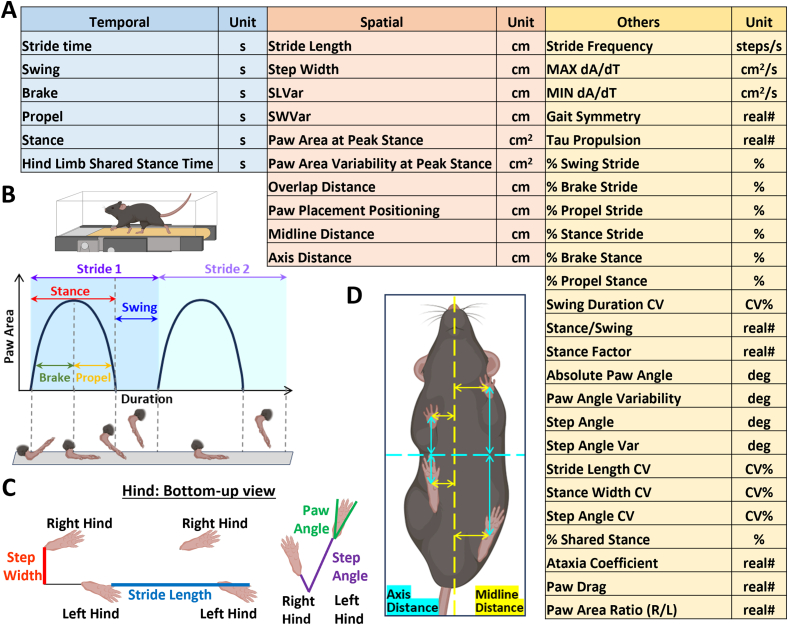
Gait analysis measures temporal, spatial and other key parameters during motion. (A) Gait parameters are categorized into three groups. Temporal outputs reflected the durations of a complete gait cycle or a step (B). Spatial outputs reflected the distance between paw and body centroids during walking or running (C and D). Others included a group of derived outputs, such as stride frequency, MAX/MIN dA/dT, duration percentages (%swing stride, %brake stride, %propel stride, %stance stride, %brake stance, %propel stance, %shared stance), ratios (Stance/swing, stance factor, gait symmetry, tau-propulsion, ataxia coefficient, paw drag, paw area ratio), angle (paw angle/variability, step angle/variability), coefficient of variation (stride length CV, step width CV, step angle CV, swing duration CV). Although all limbs were analyzed, the hindlimbs data was used due to their major functions in support and driving force.

Gait analysis was conducted using the DigiGait Imaging System (Mouse Specifics, Inc., MA, US). This equipment allows the mouse to run on a motorized treadmill with a high-speed digital camera beneath the belt to record a video of the run (Fig. 1). Each video was analyzed through the DigiGait Analysis software. The image data were extracted and analyzed frame by frame. Videos greater than 3 s offered at least 10 gait cycles to minimize the gait variability. Last, gait parameters were pooled together for hindlimbs due to their main function in support and driving force of the run. More details are provided in Supp Method.

To convert the gait output parameters into gait scores, the percentage changes comparing OA group to the control group were used as the basis of the gait scoring system. First, the means are calculated for each group. Comparing OA and control group means (ctrl(mean)), an increasing or decreasing trend was first determined to be associated with each OA gait abnormality (Fig. 6A). For example, while stride time and stride length decrease with OA, stride frequency increases with OA. Second, individual raw data are converted into percentage changes (%Change). For stride time and stride length, the difference of the control group mean minus the individual experimental group data is divided by the control group mean (Fig. 6B, OA trend: Decrease). For stride frequency, the difference of the individual experimental group data minus the control group mean is divided by the control group mean (Fig. 6B, OA trend: Increase). Lastly, each %Change was assigned a score according to the gait score table (Fig. 6C). Once converted, different gait parameters could be compared individually or summed up to reflect the total gait changes. For COG parameters, individual gait scores were summed up for stride time, stride length and stride frequency.

**Fig. 2:**
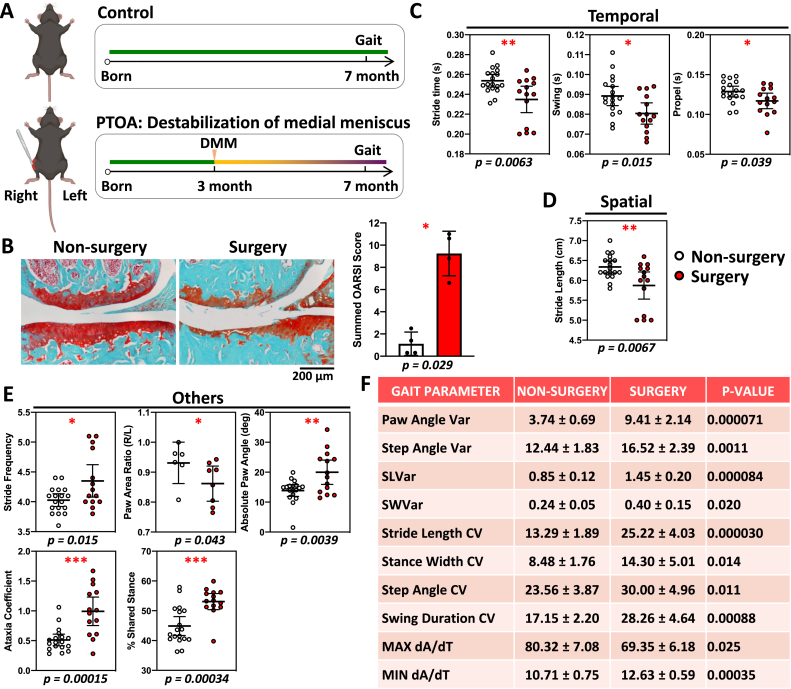
Post-traumatic osteoarthritis (PTOA) causes gait and histological changes compared to the control. Male mice were used because they were more susceptible to PTOA compared to female mice in this model. (A) Destabilization of medial meniscus (DMM) surgery was performed on 3-month-old male mice and gait data were collected at 7 months. (B) Safranin-O/fast green stained knee joint showed a loss of cartilage in the surgery group compared to the non-surgery group (left), with a significantly higher OARSI score (right). Thus, DMM model showed strong OA degeneration phenotype. (C–E) Significant gait changes were observed in temporal (C), spatial (D), and other parameters (E). ∗, p < 0.05; ∗∗, p < 0.01; ∗∗∗, p < 0.001. (F) Gait parameters demonstrated more data variations in surgery group compared to non-surgery group, which had increased variations in paw angle, step angle, stride length, stride width, step width, swing duration, and MAX/MIN dA/dT.

**Fig. 3:**
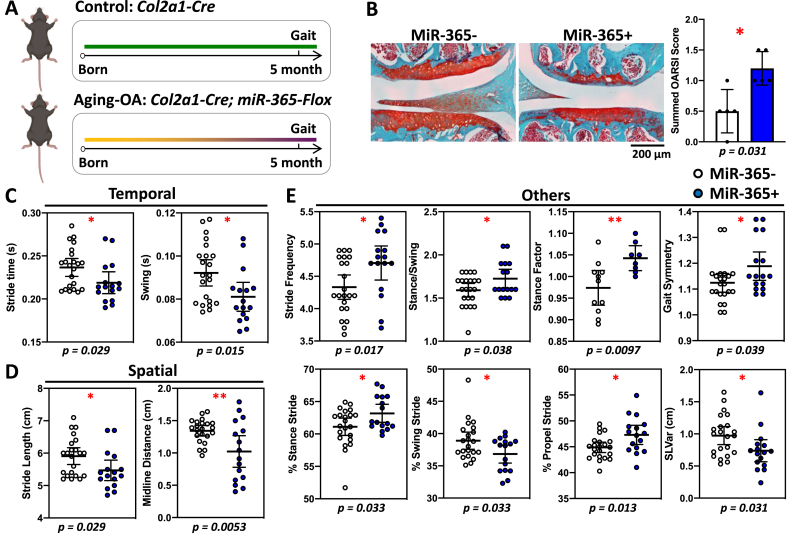
Aging-associated osteoarthritis (aging-OA) causes gait and histological changes in female transgenic miR-365 mice at 5-month of age. MiR-365 was a stress responsive microRNA, whose expression was triggered by mechanical or inflammatory stress. The transgenic mice over-expressed miR-365 in a cartilage specific manner, which induced early onset of OA pathogenesis. (A) Female miR-365 transgenic mice developed early OA at 5-month-old and gait data were collected. (B) Safranin-O/fast green stained knee joint showed a loss of cartilage in the miR-365+ group compared to the control miR-365- group (left), with a significantly higher OARSI score (right). (C–E) Significant gait changes were observed in temporal (C), spatial (D), and other parameters (E). ∗, p < 0.05; ∗∗, p < 0.01.

**Fig. 4:**
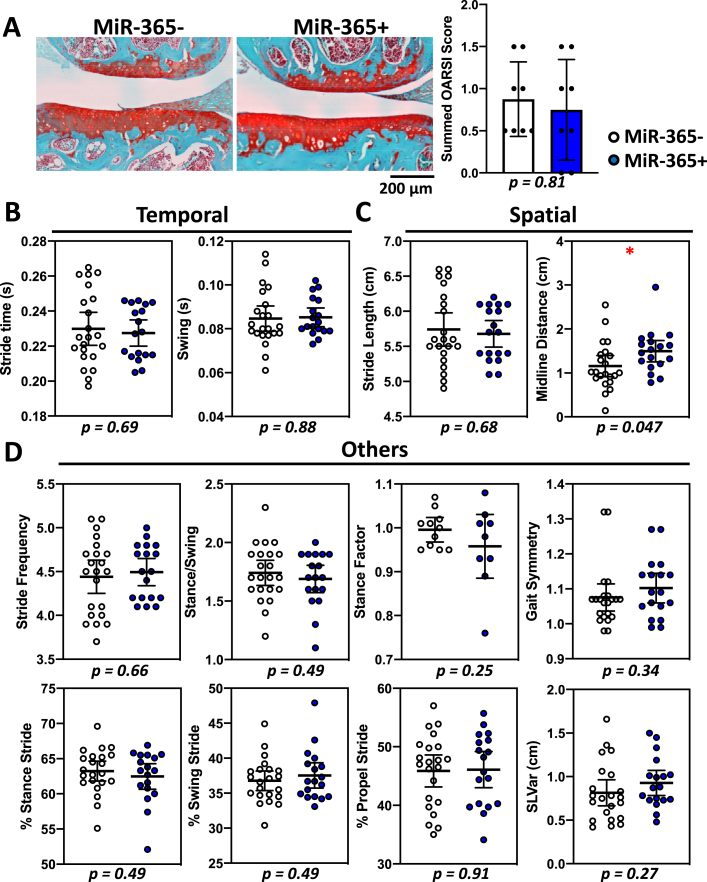
Male transgenic miR-365 mice do not develop OA or show gait changes at 5-month of age. (A) Safranin-O/fast green stained knee joint showed no obvious difference in articular cartilage between male control miR-365- and male miR-365+ groups (left), with similar OARSI scores (right). (B–D) When compared to significant gait parameters in female miR-365 mice, male miR-365 showed no difference in most of the temporal (B), spatial (C) and other parameters (E), with the only exception of midline distance in spatial parameters. ∗, p < 0.05.

**Fig. 5:**
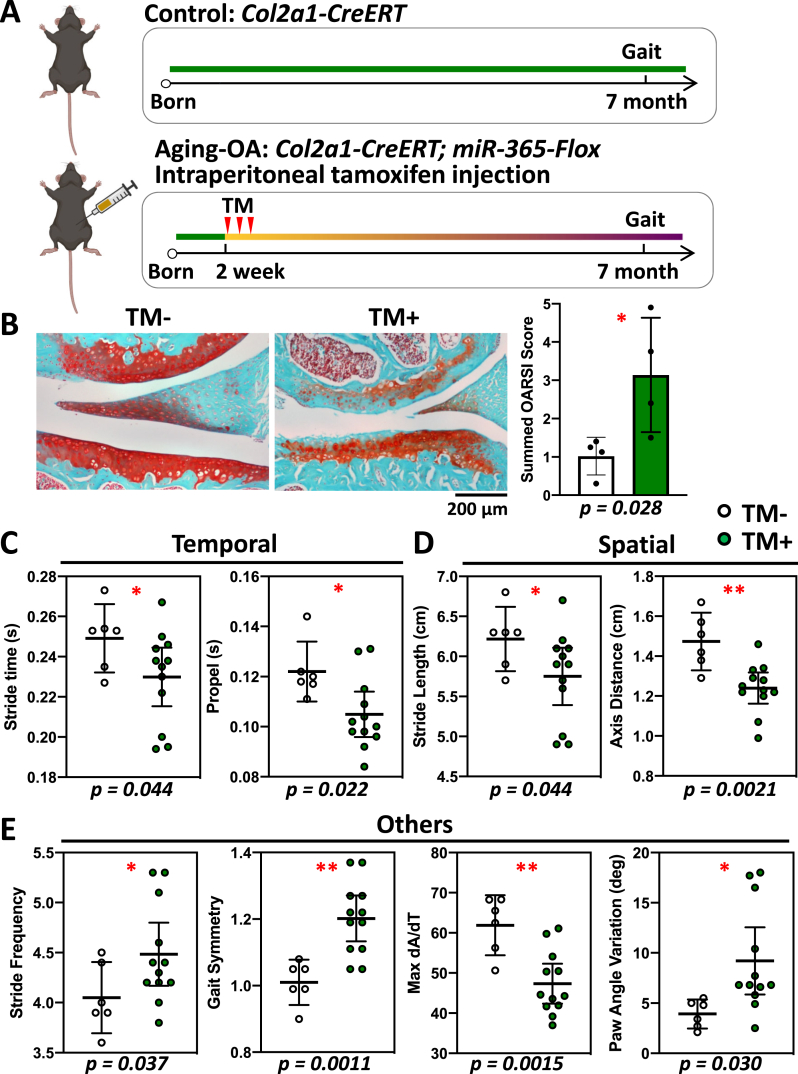
Inducible aging-OA mice also show gait and histological changes in female at 7-month of age. Tamoxifen (TM) was used for inducing miR-365 transgene expression in the inducible OA model. Although there are potential effects of tamoxifen on bone and pain, there are common gait changes associated with OA in both aging OA models regardless of tamoxifen induction. This inducible model expressed high levels of miR-365 shortly after tamoxifen injection, which gradually decreased throughout time. (A) Female inducible miR-365 mice were treated with tamoxifen through intraperitoneal injection at 2-week-old and gait data were collected at 7 months. (B) Safranin-O/fast green stained knee joint showed a loss of cartilage in the TM + group compared to the control TM-group (left), with a significantly higher OARSI score (right). (C–E) Significant gait changes were observed in temporal (C), spatial (D), and other parameters (E). ∗, p < 0.05; ∗∗, p < 0.01.

**Fig. 6:**
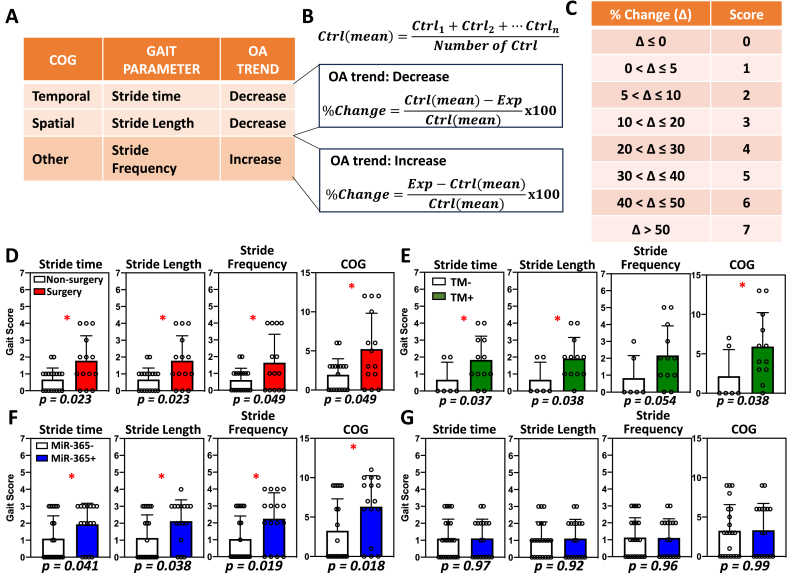
Gait parameters can be assessed through a quantitative gait scoring system. (A) Three common gait parameters (COG) showed consistent trends in all OA models tested, with stride time and stride length increasing, and stride frequency decreasing. (B) A quantitative method based on the changing trend converted raw gait data into percent changes using to a control group. (C) Gait scoring chart assigned a score according to percent change. (D–G) Converted gait scores compared the individual gait parameters and summed COG for PTOA mice (D), miR-365 aging-OA for female (F) and male (G) mice, and inducible aging-OA mice (E). ∗, p < 0.05.

### 2.3 Histology, microscopy and OARSI scoring

Detailed methods were provided in Supp Method. Briefly, mice were euthanized at 3-month and 5-month-old for miR-365 transgenic aging-OA model, and at 7-month-old for DMM PTOA models and miR-365 inducible (TM) models, as well as their age- and sex-matched control groups. Mouse knee joint images were captured on a Nikon Eclipse 90i Digital Imaging System with a 10X magnification. OA severity was quantified in the Safranin-O-stained knee joint images following the OARSI scoring system as previously described [16].

### 2.4 Statistics

Analyses were conducted using GraphPad Prism 8.0. Data are presented as means ± 95% confidence interval. All 41 gait variables were extracted from the video recording of a single test in each mouse. Individual t-tests were applied for all gait variables. Student's t-test was used to compare the differences between the control (wildtype) and experimental (OA) groups. For OARSI score and gait score comparisons, Mann-Whitney *U* test was used for p values. Differences were considered significant with P < 0.05.

## 3 Results

### 3.1 Categorizing gait analysis outputs by temporal, spatial, and other gait parameters

Gait analysis generated forty-one output parameters based on the motion video, which include various aspects of gait (Fig. 1A). As the first step of analysis, we categorized them into three main groups, Temporal, Spatial, and Others (Fig. 1). Detailed definition of each parameter is explained in Supplemental Table 1.

### 3.2 Identifying gait changes associated with DMM mice (PTOA model)

To assess gait changes in different OA models, we first examined PTOA using a surgical mouse model with significant cartilage degradation (Fig. 2A and B). PTOA resulted in shorter stride and swing times, as well as a decrease in the propel time where the foot is lifting off the ground faster (Fig. 2C). This indicated that the paws of PTOA mice preferred less time on the ground in response to DMM. Spatial gait changes showed a reduction in stride length (Fig. 2D), which correlated with a decrease of stride time. Stride frequency, paw angle, ataxia coefficient, and % shared stance also increased, whereas paw area ratio decreased (Fig. 2E). In addition, the paw area ratio decreased to less than 1 with the right surgical limb having less paw contact area with the ground compared to the non-surgery group. PTOA alters ataxia coefficient between left and right limbs in response to the DMM in the right limb. Thus, PTOA caused motion defects in each of the three gait categories and an imbalance between the injured right limb and uninjured left limb, which were characteristic gait parameters for PTOA (Fig. 2E andF).

### 3.3 Identifying gait changes associated with miR-365 transgenic mice (aging-OA model)

We examined the gait characteristics in an aging-OA model using miR-365 transgenic mice [Fig. 3A, 15]. Female miR-365 overexpressing mice (miR-365+) showed a significant loss of cartilage and an increase in the OARSI scores compared to the control mice (miR-365-) at 5 months of age (Fig. 3B). This indicated that the female miR-365 mice had an early onset of pathogenesis. Temporal gait changes included decreases in stride and swing times in female miR-365+ mice (Fig. 3C), similar to PTOA mice (Fig. 2C). Spatial gait changes included decreases in stride length and midline distance (Fig. 3D). In the Others category, female miR-365+ mice showed increases in stride frequency, stance/swing, stance factor, gait symmetry deviation, % stance stride, % propel stride, and decreases in % swing stride, and SLVar (Fig. 3E). Thus, aging-OA mice shared several common gait characteristics with PTOA mice including decreases in stride time and stride length, and an increase in stride frequency. Aging-OA mice also presented unique gait parameters including changes of gait symmetry, which suggested an imbalance between the forelimbs and hindlimbs.

In contrast, there was no obvious cartilage defects in knee histology or OARSI scores of male miR-365+ mice compared to miR-365- mice at 5-month of age (Fig. 4A). Male miR-365+ mice did not show any gait changes as seen in female miR-365+ mice (Fig. 4B–D). It had an increase in the spatial parameter midline distance, which was the opposite to female miR-365 mice (Fig. 4C). Thus, gait changes were consistent with the pathohistological analysis. It also suggested sexual dimorphism of OA pathogenesis, with prevalent aging-OA progression in female miR-365 mice.

### 3.4 Inducing miR-365 transiently caused early OA phenotype and gait changes in female mice

To determine whether aging-OA could be induced by a transient period of stress, we overexpressed miR-365 in a short period of time using an inducible mouse model (Fig. 5A). The TM-induced miR-365 overexpression led to early onset of OA with loss of cartilage and an increase in OARSI scores at 7-month of age in female mice (Fig. 5B). Temporal gait changes included decreases in stride and propel durations (Fig. 5C). Spatial gait changes included decreases in stride length and axis distance (Fig. 5D). We also observed increases in stride frequency, gait symmetry deviation and paw angle variation, and decreases in max dA/dT (Fig. 5E). These gait changes in the inducible miR-365 models were consistent with those observed in the life-long miR-365 transgenic mice. In contrast, no defects in the knee cartilage were observed in male TM+ mice, which had a comparable OARSI score as the TM- mice (Supp Fig. 2A). Stride time, stride length, axis distance, stride frequency and gait symmetry were not different between male TM+ and TM- mice (Supp Fig. 2B–D). Thus, there was sexual dimorphism in the transient stress induced OA model with female more susceptible to OA onset and development.

### 3.5 Developing the OA gait grading system to quantify common OA gait in mice

Although the three OA models examined so far were quite different in the induction and timing of OA onset, there were common OA gait parameters shared among different OA types. These included a decrease of stride time in the temporal parameters, a decrease of stride length in the spatial parameters, and an increase of stride frequency in the others category (Fig. 6A). Together, they were common OA gait (COG) parameters. However, these parameters belonged to different categories with different units. There has been no method to collectively compare all gait changes associated with OA phenotypes. Therefore, we developed a quantitative gait grading system that allowed a combined assessment of COG parameters (Fig. 6B and C).

Using the gait scoring systems, we examined the COG parameters in different OA models. For the PTOA DMM mice, there were increasing gait scores for stride time, stride length, and stride frequency individually, which resulted in a significant increase of summed gait scores for the COG (Fig. 6D). For both the inducible (TM) and transgenic aging-OA mice, there were increasing gait scores for stride time, stride length, and stride frequency individually, as well as the summed gait scores for the COG in the female mice (Fig. 6, E and F), but not in the male mice (Fig. 6G and Supp Fig. 2E). Thus, the COG scores adequately reflected the OA-associated gait changes in each group, consistent with the histopathological changes by the OARSI grading system. However, no significant correlation was found between COG and OARSI scores using Spearman correlation (coefficient = 0.1077, p-value = 0.71).

### 3.6 Detecting early OA phenotypes with the OA gait grading system

**Fig. 7:**
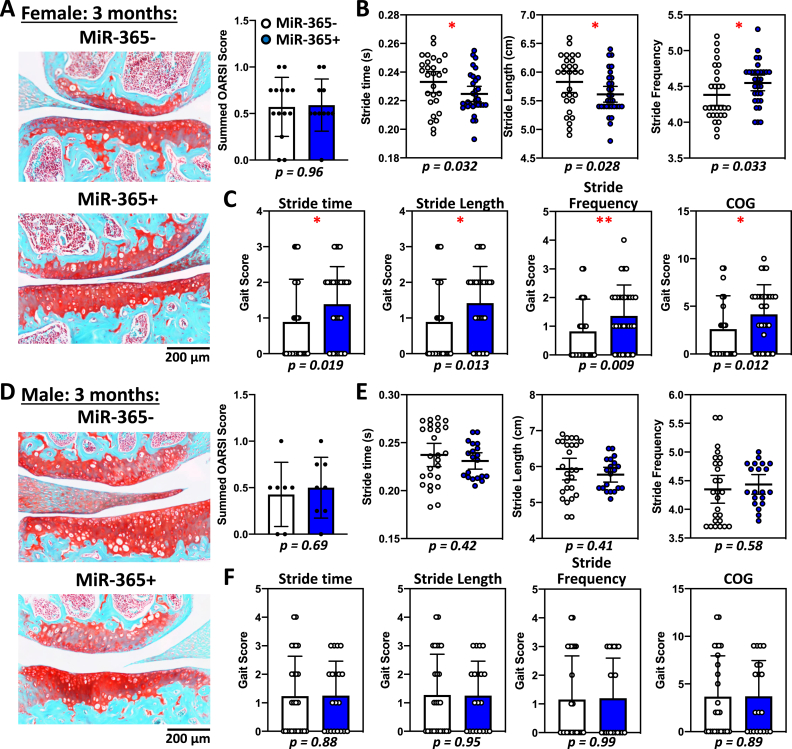
Gait changes in female aging-OA mice occur before histological changes, but not in male aging-OA mice of the same age. (A) Female miR-365 transgenic mice at 3-month-old showed no obvious histological changes in safranin-O/fast green stained articular cartilage (left) and OARSI score (right). (B) Gait changes show decreases in stride time and stride length, and increases in stride frequency. (C) Converted gait scores were significantly higher in female miR-365+ mice for the common three gait parameters and the summed COG. ∗, *p* < 0.05; ∗∗, p < 0.01. (D) Male miR-365 transgenic mice at 3-month-old showed no obvious histological changes in articular cartilage (left) and OARSI score (right). (E) No significant differences were observed in stride time, stride length, and stride frequency. (F) Converted gait scores were not significantly different between male miR-365- and miR-365+ mice.

Next, we tested whether the COG grading system could detect early OA phenotypes. Although the female transgenic miR-365 mice had shown a significant loss of articular cartilage at 5-month of age (Fig. 3B), there was no difference in cartilage histology between female miR-365+ and miR-365- mice at 3-month of age (Fig. 7A). However, gait analysis indicated decreases in stride time, stride length, and an increase in stride frequency in female miR-365+ mice at 3-month of age (Fig. 7B). Gait scores for individual parameters and the summed COG score were significantly higher in female miR-365+ mice (Fig. 7C), although there is no difference in the OARSI score (Fig. 7A). Conversely, male miR-365+ mice did not develop OA histopathology at either 3-month or 5-month of age (Fig. 4, Fig. 7D), indicating the absence of OA pathogenesis during this period. Gait analysis did not detect any COG changes at either 3- or 5-month of age in male miR-365+ mice (Figs 4, B–D, and 7, E and F), in contrast to the female miR-365+ mice. These data indicated that gait changes might precede cartilage histological changes during OA pathogenesis, and the COG grading system was more sensitive to detecting OA onset and early stages than histopathological grading system in some aging-OA models.

### 3.7 Quantifying disease progression with the OA gait grading system during OA pathogenesis

**Fig. 8:**
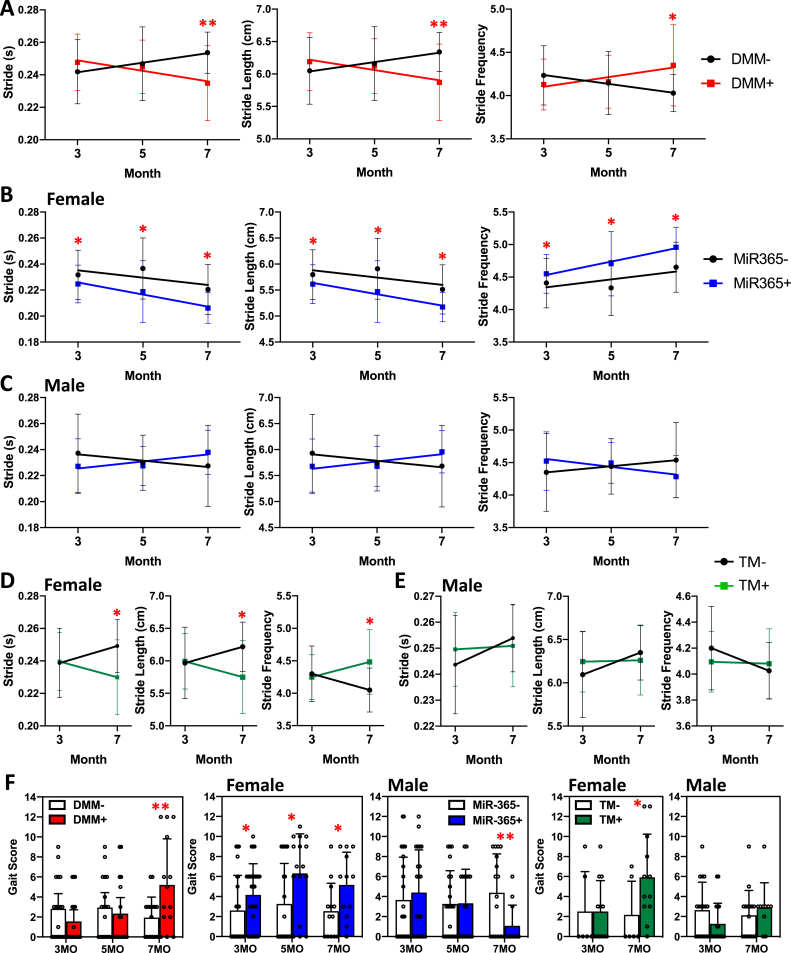
Gait changes over time for the common gait parameters in PTOA and aging-OA mice. (A–E) Stride time, stride length, and stride frequency for PTOA (A), female (B) and male (C) transgenic miR-365 aging-OA, female (D) and male (E) inducible TM aging-OA between 3-month and 7-month of age. Control and OA groups were compared at the same age. (F) Summed gait score for stride time, stride length and stride frequency at different timepoints in the OA models. Control and OA groups were compared at the same age. ∗, p < 0.05; ∗∗, p < 0.01.

To understand the effect of age on gait, we examined the COG trends in PTOA, aging-OA, and inducible aging-OA mice between 3- and 7-month of age. In all three cases including male DMM+ mice, female miR-365+ constitutive and TM+ inducible mice, stride time and stride length were decreased, and stride frequency was increased (Fig. 8A, B, D). It resulted in a significant increase in the COG score for DMM+ and female TM+ mice at 7-month of age (Fig. 8F). In contrast, the COG scores were consistently increased at 3-, 5-, and 7-month of age in the female miR-365+ constitutive expression mice (Fig. 8F). Thus, the COG grading system revealed a later onset and disease progression in the inducible OA mice than the transgenic OA mice. In contrast, at 7-month of age, there was no COG score increase in the male TM+ mice. The COG score was even decreased in the male miR-365+ mice (Fig. 8F), revealing an unexpected strengthening of the gait parameters in the 7-month-old male miR-365+ mice.

## 4 Discussion

In this study, we characterized gait behavior in multiple OA mouse models and developed an OA Gait grading system that captured OA pathogenesis quantitatively. We discovered common OA gait (COG) parameters including stride time, stride length, and stride frequency. Gait changes also reflected the sex dimorphism associated with OA, with female mice having more pronounced gait deficiency compared to male mice during aging-OA pathogenesis. These data revealed gait changes common and specific for PTOA vs. aging-OA. They indicated the advantage of using gait as a sensitive tool for early diagnosis of OA.

Gait changes were characterized for the PTOA model (DMM+) and aging-OA models (TM+ and miR-365+) at different stages of knee OA. The DMM model is a common surgical model for PTOA with extensive published literature [11] while the miR-365 transgenic mouse models mimic early onset of aging-OA [18]. In both PTOA and aging-OA, stride time and stride length were decreased, whereas stride frequency was increased. Based on the gait data, a quantitative grading system was established for the overall evaluation of gait changes combining multiple parameters (Fig. 6A–C). The COG scores were significantly higher for both PTOA and aging-OA groups when OA progression was observed, such as the female miR-365+ mice (Fig. 6D–F). On the other hand, there was no significant increase of the COG scores when OA progression was not observed, such as in the male miR-365+ mice (Fig. 6G).

In addition to the COG, we also identified specific OA gait associated with PTOA and aging-OA. Proper balance is also another key factor involved in gait analysis and can be generalized as *symmetry*. DigiGait has two types of symmetry. The first type compares the left and right balance, usually seen in unilateral knee OA. For example, “paw area ratio (R/L)” is an indicator of such balance change. If gait is normal, the paw area in contact with the ground will be the same with a ratio of 1. If injured in one joint, the injured limb will decrease contact with the ground due to pain caused by OA inflammation. Thus, it caused a decrease in paw area ratio (Fig. 2E). Javaheri et al. had also identified L/R asymmetry in OA-prone STR/Ort mice, which was positively correlated with OA severity [29,30]. The second type of symmetry compares the balance between forelimbs and hindlimbs, such as “gait symmetry” (the ratio of forelimb stride frequency over hindlimb stride frequency). Aging-OA models showed significant increases in gait asymmetry for both female constitutive and inducible miR-365 transgenic mice (Fig. 3, Fig. 5E). This suggests an imbalance between the motions in forelimbs and hindlimbs. Both types of gait asymmetry affect gait cycles, but the left and right symmetry is more relevant in humans. Studies comparing asymmetries in foot posture and gait showed that unilateral knee OA had more severe asymmetry compared to bilateral knee OA [31,32]. Although both had foot posture asymmetry and gait asymmetry, unilateral knee OA patients had higher risk of falling due to unequal distribution of body weight and foot pressure [31,32]. Therefore, gait asymmetry is a reasonable indicator of gait abnormality and a key factor in OA assessment.

Gait changes were also able to reflect the OA sex dimorphism in aging-OA models, where female aging-OA mice showed significant gait changes, but the male aging-OA mice did not at the same age. The difference observed in female and male gait analysis supports the fact that female has a higher risk for aging-OA [4,33,34]. Our aging-OA results in this study not only showed trends in gait parameter changes but also examined sex dimorphism in OA through a gait perspective. Most interestingly, when female was gradually developing OA associated gait changes under miR-365 overexpression (Fig. 8, B and D), male showed opposite gait changing trends under miR-365 overexpression as they aged (Fig. 8, C and E). Gait scores for male miR-365+ were even lower compared to male miR-365- control at 7-month-old (Fig. 8F), suggesting that an encounter of stress at an early age may be beneficial and improve gait performance in male.

We found that gait changes occurred in female aging-OA mice at 3-month of age before histological changes were observed (Fig. 7). This may explain the lack of correlation between COG and OARSI scores when gait abnormality occurred before histological changes. This suggests that gait changes may be more sensitive than the traditional histology method in some aging-OA models. Although not universal in all OA models, this high gait sensitivity to locomotion abnormalities still shows potential for further investigation in its use as early detection methods for disease onset. Based on the findings in this study and other studies, there are many advantages to the application of gait analysis and gait scoring system in research. First, gait analysis is a behavioral test that does not cause injury or require end-point data collection. In fact, it is a convenient experiment that can be repeated and performed at multiple timepoints throughout the lifespan. Second, the gait scoring system allows for the evaluation of multiple gait parameters together. This method enables unbiased analysis of gait parameters in different categories, which can be compared separately or in a summed way. Meanwhile, gait scores offer a strong degree of freedom to select relevant parameters both common and unique to a model for the most accurate comparisons. Third, raw gait data converted into gait scores remove the fundamental development differences between female and male, such as body weight, length and width. Gait parameters in spatial and temporal categories are strongly affected by the body sizes of the subjects, thus showing significant differences between female and male irrelevant of OA status. By converting these gait data into percent changes, gait scores can be compared between sex and OA status.

The most fundamental cause for gait abnormalities lies in the structural changes associated with musculoskeletal disease progression. Direct degradation of the articular cartilage structures in OA and rheumatoid arthritis has strong impacts on various gait parameters, and is often exacerbated by increased inflammation in the knee joint [[22], [23], [24], [25],^35^]. Other musculoskeletal diseases affecting joint muscle (muscular dystrophy) or synovium (diarthrodial joint arthropathy) also lead to gait abnormalities, with high similarities in changing gait parameters [36,37]. Besides the direct pathological changes in musculoskeletal structures, neurodegenerative diseases have been shown to indirectly affect gait behavior. For example, Alzheimer's disease [[38], [39], [40], [41], [42]], Parkingson's disease [38,43,44], and Huntington's disease [38,45] can cause significant gait changes in stride length and stride frequency. Neurological disorders impact gait behavior through its neuronal signaling control of motor movement in the limbs and motion coordination. Thus, treatments for musculoskeletal and neurodegenerative diseases can reverse the gait abnormalities associated with disease pathology. Rescue of structural degradation and reduction in disease phenotypes often lead to improved gait parameters in both animal models and clinical cases [35,[46], [47], [48], [49]].

Although gait analysis is easy to use, there are some aspects that need careful consideration. First, sex must be considered when performing gait analysis. Due to the development differences (Supp Fig. 1, B–I), mixing female and male gait will greatly impact the results. Second, it is necessary to have proper control groups. Matching age, sex, and wildtype strain are important for normalization and gait score conversion. A limitation with gait analysis is the use of different speed across multiple studies. Velocity matching is critical in each study between the control and experimental groups. By converting the raw gait parameters into gait scores, it may minimize the differences in raw data caused by various speeds and allow for comparisons across different studies. Another limitation is the direct comparison between animal gait and clinical gait. Due to the quadruped vs. biped gait, data inference needs careful examination and validation. Potentials of gait compensation by the contralateral limbs may play a role in gait differences between animal and human. Due to our focus on gait, we did not examine bone or synovium structures in detail. However, the overall environmental changes in the OA knee joint may contribute to gait abnormality. With the above taken into consideration, gait analysis can be a powerful tool and provide in depth data associated with disease onset and progression.

In summary, we identified three common gait parameters that have consistent changing trends associated with OA pathology and found evidence that gait changes may be sensitive to early stage of OA development. Meanwhile, we developed a quantitative and non-biased OA Gait scoring system to quantify multiple relevant gait parameters. Most importantly, analysis of these gait abnormalities provides an effective way of detecting early onset of diseases affecting mobility. The COG represents the first important step to develop a uniformed gait score system to detect OA changes, which will be subject to further validation, revision and improvement.

## Author contributions

Y.L. and Q.C. conceptualized and designed the study, Y.L., J.D. and A.K. conducted experiments, Y.L. and A.K. analyzed the data. Y.L., G.J. and Q.C. wrote the manuscript. All authors reviewed and edited the manuscript.

## Role of the funding source

This study was supported by NIH/NIGMS (P30GM122732), NIH/NIAMS (R61/R33 AR076807), and NIH/NIA (1R01AG080141) to QC and by a pilot grant and a research project lead grant from NIH/NIGMS (5P20GM109035-08) to YL.

## Conflict of interest

None of the authors have competing interests regarding this work.

## Appendix A Supplementary data

Multimedia component 2 Multimedia component 2

Multimedia component 3 Supplemental Figure 1. Developmental parameters for PTOA and aging-OA mice. (A) Summary of control and OA mouse groups. (B) Body length and width are measured during gait analysis. (C-I) Comparisons of mouse body weight, body length and width for PTOA (C), female (D, F) and male (E, G) miR-365 aging-OA at 5-month (D-E) and 3-month (F-G) of age, female (H) and male (I) inducible TM aging-OA mice. DMM surgery did not affect the weight and body length of the mice but caused an increase in body width. There is no difference in weight, body length and width between miR-365- and miR-365+ mice. TM injection did not affect the weight and body length of mice but caused an increase in body width in female TM+ mice. ∗∗, *p* < 0.01. Supplemental Figure 2. Male inducible aging-OA mice does not show OA phenotypes or common gait changes, but with some other gait differences. (A) Safranin-O/fast green stained knee joint showed no obvious loss of cartilage in the male TM+ group compared to the control TM- group (left), with a slightly lower OARSI score (right). (B-D) Gait parameters were compared between male TM- and TM+ in temporal (B), spatial (C), and other relevant parameters (D). (E) Individual or summed gait scores show no difference in male inducible aging-OA mice. ∗∗, *p* < 0.01; ∗∗∗, *p* < 0.001. Multimedia component 3
