# Supplementary material for: The common and specific osteoarthritis gait characteristics: A quantitative grading system for OA associated gait changes in mice
Source: Osteoarthr Cartil Open. 2026 Feb 18;8(2):100757. doi: 10.1016/j.ocarto.2026.100757 (PMC12972523; doi:10.1016/j.ocarto.2026.100757)
Supplement: Multimedia component 1 [file mmc1.docx]

**Supplemental Methods**

OA mouse models

For the PTOA model, knee joint instability was induced by the destabilization of medial meniscus (DMM) surgery on the right hindlimb of male *Col2a1-CreER^T^* mice. The *Col2a1-CreER^T^* mice were purchased from The Jackson Laboratory (Strain: 006774). At 3 months of age, mice were anesthetized and surgically disconnected the anterior attachment of the medial meniscus to the tibial plateau (DMM+)^1, 2^. A separate group of unoperated mice were used as the non-surgery group (DMM-). For aseptic preparation, we sterilized all instruments for surgery using high-pressure autoclave or use sterile disposable surgical equipment. All personnel involved in the surgery had surgeon caps, face masks, sterile drapes and gloves throughout the procedure. The mice were anesthetized using isoflurane via inhalation. The skin and soft tissue were cut in a vertical direction along the limb next to the medial meniscus in 0.5 cm length. A small blade was then carefully inserted into the joint cavity to cut the medial meniscotibial ligament. Subcuticular suturing technique was used with absorbable vicryl sutures to close the wound. Buprenorphine was given at 0.03 mg/kg twice a day for three consecutive days to reduce pain and prevent wound scratching by the mice.

For the aging OA models, cartilage-specific miR-365 overexpression and inducible transgenic mouse models were used, including both female and male mice. *MiR-365-flox* mice in C57BL/6 background were generated at Brown University Mouse Transgenic and Gene Targeting Facility as previously described^3^. Overexpression of the stress responsive miR-365 in the articular cartilage tissues was achieved by crossing *miR-365-flox* mice with *Col2a1-Cre* mice^4^, resulting in *miR-365-flox*+; *Col2a1-Cre*+ transgenic mice (miR-365+). The *Col2a1-Cre* mice were purchased from The Jackson Laboratory (Strain: 003554). Its littermate *miR-365-flox*-; *Col2a1-Cre*+ mice were used as the control group (miR-365-). The inducible miR-365 model was achieved by crossing *miR-365-flox* mice with *Col2a1-CreER^T^* mice^5^, which resulted in *miR-365-flox*+; *Col2a1-CreER^T^* + mice. They overexpressed miR-365 after tamoxifen-dependent activation (TM+). Its littermate *miR-365-flox*-; *Col2a1-CreER^T^*+ mice were used as the control group (TM-). Tamoxifen (T5648, Sigma-Aldrich, MA, US) was dissolved in corn oil (c8267, Sigma, MA, US) at a concentration of 50 mg/ml by shaking overnight at 37°C. Animals received tamoxifen every other day at 125 mg/kg body weight intraperitoneally for 3 times at 2 weeks of age. The aging-OA models were euthanized at OA onset when we see histological cartilage degradation in OA females, which is 5 months for *Col2-Cre;miR-365* and 7 months for *Col2-CreERT;miR-365*. The transgenic aging-OA model was euthanized at an earlier timepoint (3 months) due to our observation in gait changes at this age. Therefore, we examined gait changes in relation to histology in 3-month old.

Gait analysis

Gait analysis was conducted using the DigiGait Imaging System (Mouse Specifics, Inc., MA, US). This equipment allows the mouse to run on a motorized treadmill with a high-speed digital camera beneath the belt to record a video of the run (**Fig 1**). Before each run, the mouse was first weighed and placed onto the treadmill. The treadmill was turned on at a set speed first for a few seconds for the mouse to adjust, and then the video recording started. To collect sufficient gait data, the videos were taken for a short 3-5 seconds depending on the running ability of the mouse. Based on preliminary results in the lab, the treadmill speed for this experiment is 25 cm/s with no inclination. This speed is appropriate for our study design because it can keep the adult mice in running posture. If the speed is too low, some mice will walk/run and halt at their own will or have chances to turn around to the rear end. Such raw data had patterns similar to diseased data, thus making it hard to distinguish between control and experiment groups. It is critical to identify the best testing speed that allows the mice to run at a relatively constant pace.

Once the video data were collected, each video was analyzed through the DigiGait Analysis software. Post-processing of the video is based on digitally captured paw prints as the mice run. The image data were extracted and analyzed frame by frame. Initial analysis required some manual corrections to adjust the signals picked up for each paw print. Noises and background signals from the fur, the tail, and the mouth were removed before analyzing paw signals. The paw-belt contact areas for each limb were tracked and plotted into curves for generating the gait parameters. Videos greater than 3 seconds offered at least 10 gait cycles to minimize the gait variability. Last, gait data for temporal, spatial, and other gait parameters derived were pooled together for hindlimbs due to their main function in support and driving force of the run.

Safranin-O histology

Mice were euthanized at 3-month and 5-month-old for miR-365 transgenic aging-OA model, and at 7-month-old for DMM PTOA models and miR-365 inducible (TM) models, as well as their age- and sex-matched control groups. Both left and right knee joints were fixed in 10% formalin (HTS01128-4L, Millipore Sigma, MA, US) for 24h at room temperature, decalcified in 14% EDTA, pH 7.4 (BM-150A, Boston BioProducts, MA, US) for 10 days at room temperature, and paraffin embedded. The paraffin blocks were sectioned into 3 um frontal section samples and mounted onto slides. Sample slides were processed with xylene, and rehydrated through sequential incubation in 100%, 95%, 70% and 50% ethanol solution. After rinsing in deionized water, samples were stained with 0.1% Safranin-O (S8884, Sigma, MA, US) and 0.4% fast green (F-7258, Sigma, MA, US) as described previously^6^. Last, stained tissues on the slide were sealed with resinous mounting medium (ACRYMOUNT SL80-4, Fisher Scientific, PA, US) for imaging and storing.

Microscopy

Mouse knee joint images were captured on a Nikon Eclipse 90i Digital Imaging System with a 10X magnification. A large scan of the whole knee section is performed by manual focusing on multiple smaller areas and stitching the fragments together. This method removed the out of focus issue caused by scanning a large area at a single focal plane. Images were exported as TIFF files and further processed using ImageJ.

OARSI scoring

To evaluate OA severity, we quantified the Safranin-O-stained knee joint images following the OARSI scoring system as previously described^7^. Briefly, we used a 0 to 6 scoring system to assess all four quadrants of the mouse knee joint in a blinded fashion. The summed OARSI score is expressed as the combination of femoral condyle and tibial plateau of the same side. Multiple evaluators (3 for PTOA, 2 for aging OA) scored the data separately and the average scores were used.

**References in Supplemental Methods**

1. Glasson SS, Blanchet TJ, Morris EA. The surgical destabilization of the medial meniscus (DMM) model of osteoarthritis in the 129/SvEv mouse. Osteoarthritis Cartilage 2007; 15: 1061-1069.

2. Botter SM, Glasson SS, Hopkins B, Clockaerts S, Weinans H, van Leeuwen JP, van Osch GJ. ADAMTS5-/- mice have less subchondral bone changes after induction of osteoarthritis through surgical instability: implications for a link between cartilage and subchondral bone changes. Osteoarthritis Cartilage 2009; 17: 636-645.

3. Yang K, Gao Y, Yang M, Xu Z, Chen Q. Creating conditional dual fluorescence labeled transgenic animals for studying function of small noncoding RNAs. Connect Tissue Res 2017; 58: 103-115.

4. Ovchinnikov DA, Deng JM, Ogunrinu G, Behringer RR. Col2a1-directed expression of Cre recombinase in differentiating chondrocytes in transgenic mice. Genesis 2000; 26: 145-146.

5. Nakamura E, Nguyen MT, Mackem S. Kinetics of tamoxifen-regulated Cre activity in mice using a cartilage-specific CreER(T) to assay temporal activity windows along the proximodistal limb skeleton. Dev Dyn 2006; 235: 2603-2612.

6. Guan YJ, Li J, Yang X, Du S, Ding J, Gao Y, et al. Evidence that miR-146a attenuates aging- and trauma-induced osteoarthritis by inhibiting Notch1, IL-6, and IL-1 mediated catabolism. Aging Cell 2018; 17: e12752.

7. Glasson SS, Chambers MG, Van Den Berg WB, Little CB. The OARSI histopathology initiative - recommendations for histological assessments of osteoarthritis in the mouse. Osteoarthritis Cartilage 2010; 18 Suppl 3: S17-23.
