# Supplementary material for: The common and specific osteoarthritis gait characteristics: A quantitative grading system for OA associated gait changes in mice
Source: Osteoarthr Cartil Open. 2026 Feb 18;8(2):100757. doi: 10.1016/j.ocarto.2026.100757 (PMC12972523; doi:10.1016/j.ocarto.2026.100757)
Supplement: Multimedia component 2 [file mmc2.docx]

**Supplemental Table 1. Definition of DigiGait parameters.**

| **Parameter** | **Unit** | **Definition** |
| --- | --- | --- |
| Temporal | | |
| Stride time* | Second | Time of one paw taking a complete step |
| Stance | Second | Time of paw contact with the ground in a stride |
| Swing | Second | Time of no paw contact with the ground in a stride |
| Brake | Second | Time of initial paw contact to maximum paw contact with the ground |
| Propel | Second | Time of maximum paw contact to lifting off the ground |
| Hind Limb Shared Stance Time | Second | Time when both hind paws in contact with the ground |
| Spatial | | |
| Stride Length | Centimeter | Distance one paw travels in a stride |
| Step Width** | Centimeter | Distance between both hind limbs |
| SLVar | Centimeter | Standard deviation of stride lengths |
| SWVar | Centimeter | Standard deviation of stride widths |
| Paw Area at Peak Stance | Square centimeter | Paw area captured at maximum stance |
| Paw Area Variability at Peak Stance | Square centimeter | Standard deviation of paw areas |
| Overlap Distance | Centimeter | Overlap distance of ipsilateral fore and hind paws |
| Paw Placement Positioning | Centimeter |  |
| Midline Distance | Centimeter | Distance between one paw and the midline of the mouse at peak stance |
| Axis Distance | Centimeter | Distance between one paw and paw centroids |
| Others | | |
| Stride Frequency | Steps/second | Number of strides per second (cadence) |
| MAX dA/dT | Square centimeter/second | Max rate of paw area in contact with belt during brake phase |
| MIN dA/dT | Square centimeter/second | Max rate of paw area in contact with belt during propel phase |
| Gait Symmetry | Real# | Ratio of forelimb stride frequency over hind limb stride frequency |
| Tau Propulsion | Real# | Time constant for gait signal decay during propel phase |
| % Swing Stride | Percentage | Percent of swing phase in a stride |
| % Brake Stride | Percentage | Percent of brake phase in a stride |
| % Propel Stride | Percentage | Percent of propel phase in a stride |
| % Stance Stride | Percentage | Percent of stance phase in a stride |
| % Brake Stance | Percentage | Percent of brake phase in a stance |
| % Propel Stance | Percentage | Percent of propel phase in a stance |
| Swing Duration CV | CV% | Ratio of standard deviation and average for swing duration |
| Stance/Swing | Real# | Ratio of stance duration to swing duration |
| Stance Factor | Real# | Ratio of left and right stance durations |
| Absolute Paw Angle | Degree | The angle of one paw stretched out during locomotion |
| Paw Angle Variability | Degree | Standard deviation of paw angles |
| Step Angle | Degree | The angle between left and right hind paws |
| Step Angle Var | Degree | Standard deviation of step angles |
| Stride Length CV | CV% | Ratio of standard deviation and average for stride length |
| Stance Width CV | CV% | Ratio of standard deviation and average for stride width |
| Step Angle CV | CV% | Ratio of standard deviation and average for step angle |
| % Shared Stance | Percentage | Percent of stance when both hind paws are in contact with the ground |
| Ataxia Coefficient | Real# | Difference of max and min stride length divided by average stride length |
| Paw Drag | Real# | Area under gait curve from full stance to toe-off |
| Paw Area Ratio (R/L) | Real# | Ratio of right paw area at peak stance over left paw area at peak stance |

*DigiGait output term is “Stride”.

**DigiGait output term is “Stance Width”.
