# Supplementary material for: The common and specific osteoarthritis gait characteristics: A quantitative grading system for OA associated gait changes in mice
Source: Osteoarthr Cartil Open. 2026 Feb 18;8(2):100757. doi: 10.1016/j.ocarto.2026.100757 (PMC12972523; doi:10.1016/j.ocarto.2026.100757)
Supplement: Multimedia component 3 — Supplemental Figure 1. Developmental parameters for PTOA and aging-OA mice. (A) Summary of control and OA mouse groups. (B) Body length and width are measured during gait analysis. (C-I) Comparisons of mouse body weight, body length and width for PTOA (C), female (D, F) and male (E, G) miR-365 aging-OA at 5-month (D-E) and 3-month (F-G) of age, female (H) and male (I) inducible TM aging-OA mice. DMM surgery did not affect the weight and body length of the mice but caused an increase in body width. There is no difference in weight, body length and width between miR-365- and miR-365+ mice. TM injection did not affect the weight and body length of mice but caused an increase in body width in female TM+ mice. ∗∗, p < 0.01. Supplemental Figure 2. Male inducible aging-OA mice does not show OA phenotypes or common gait changes, but with some other gait differences. (A) Safranin-O/fast green stained knee joint showed no obvious loss of cartilage in the male TM+ group compared to the control TM- group (left), with a slightly lower OARSI score (right). (B-D) Gait parameters were compared between male TM- and TM+ in temporal (B), spatial (C), and other relevant parameters (D). (E) Individual or summed gait scores show no difference in male inducible aging-OA mice. ∗∗, p < 0.01; ∗∗∗, p < 0.001. [file mmc3.pptx]

## Slide 1
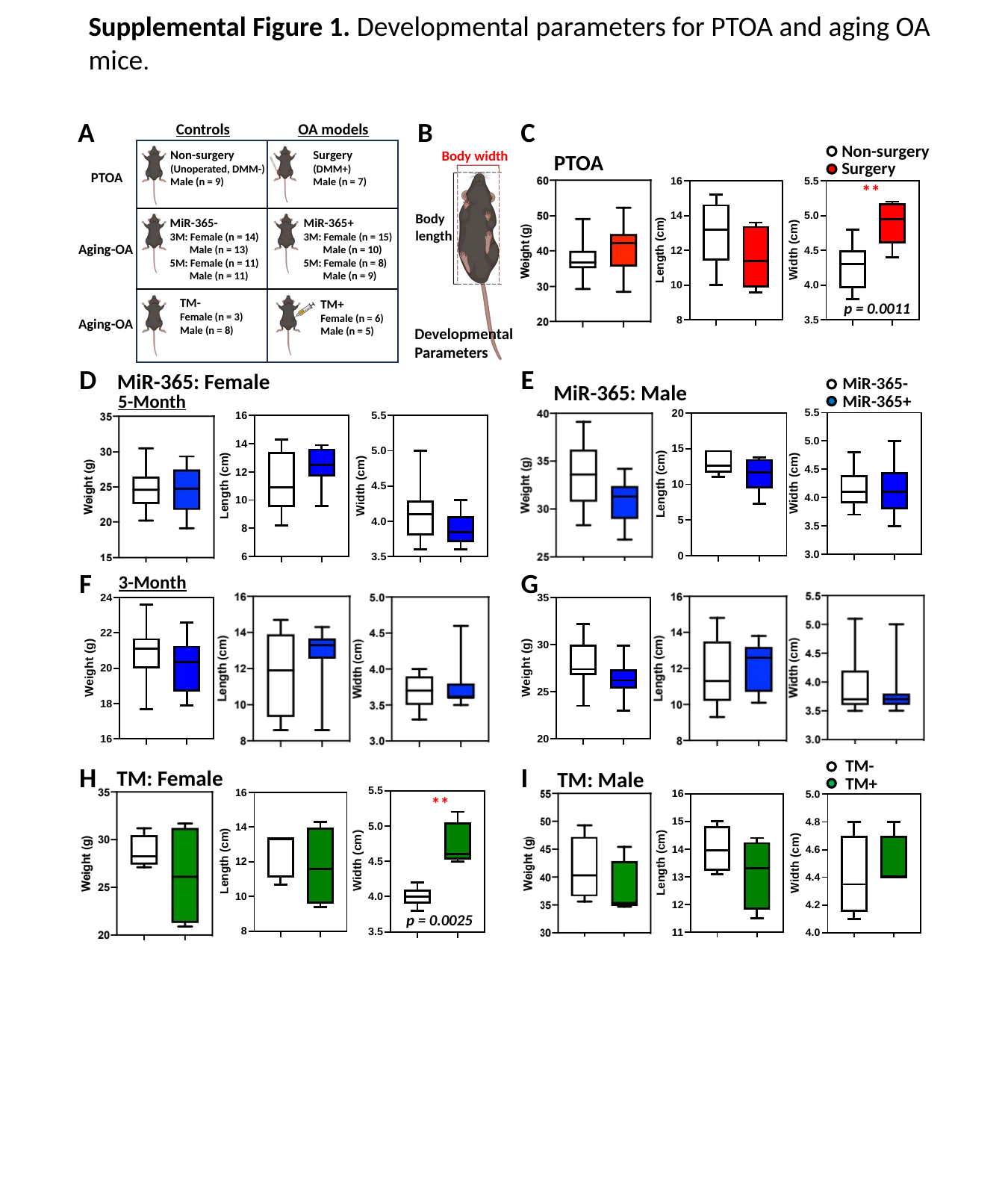

Supplemental Figure 1. Developmental parameters for PTOA and aging OA mice.
A
B
C
Controls
OA models
Non-surgery
(Unoperated, DMM-)
Male (n = 9)
Surgery
(DMM+)
Male (n = 7)
PTOA
MiR-365-
3M: Female (n = 14)
 Male (n = 13)
5M: Female (n = 11)
 Male (n = 11)
MiR-365+
3M: Female (n = 15)
 Male (n = 10)
5M: Female (n = 8)
 Male (n = 9)
Aging-OA
TM-
Female (n = 3)
Male (n = 8)
TM+
Female (n = 6)
Male (n = 5)
Aging-OA
Non-surgery
Surgery
PTOA
**
p = 0.0011
Body width
Body
length
Developmental Parameters
D
E
MiR-365: Female
MiR-365-
MiR-365+
MiR-365: Male
5-Month
F
G
3-Month
TM-
TM+
H
I
TM: Female
**
p = 0.0025
TM: Male

## Slide 2
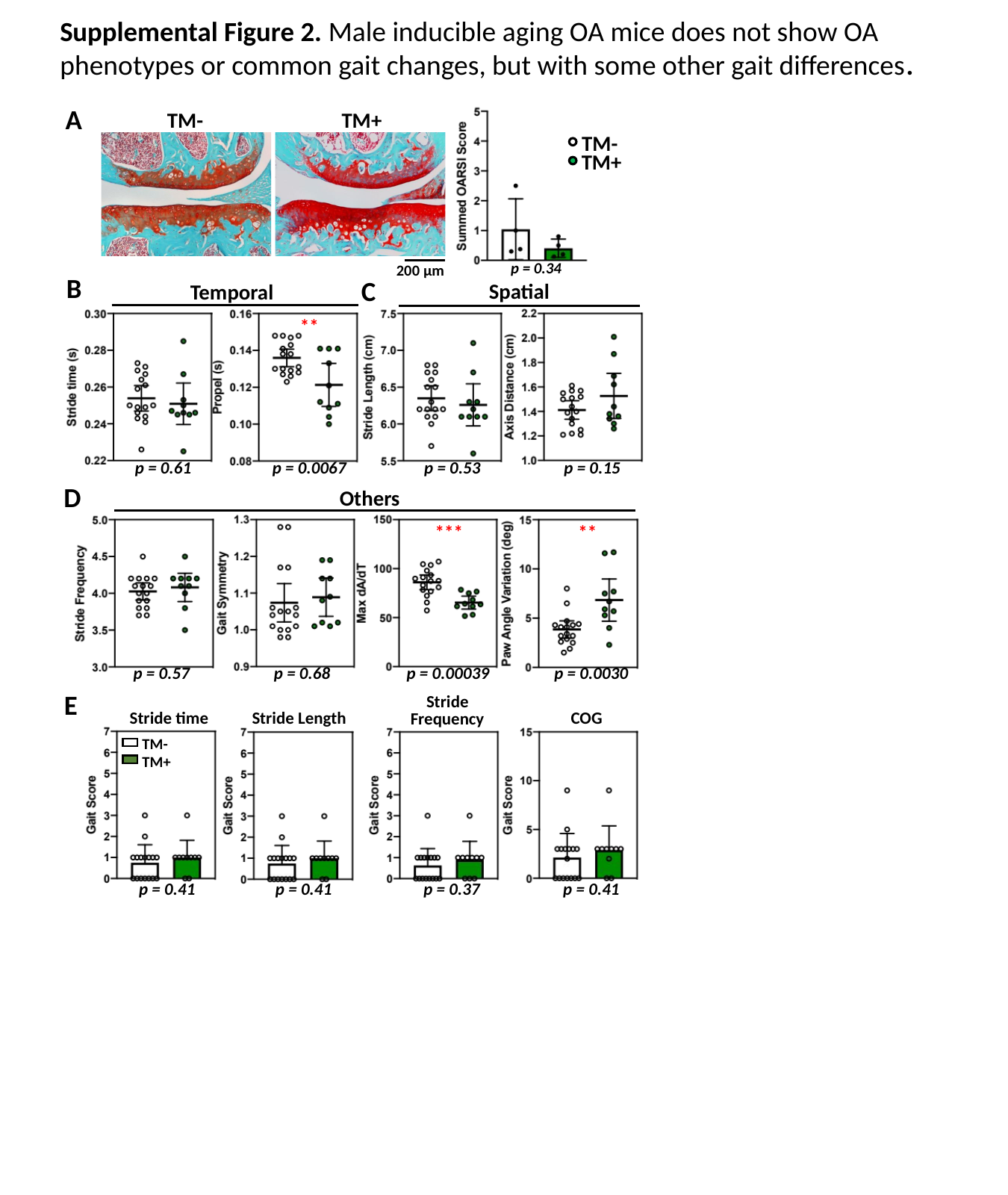

Supplemental Figure 2. Male inducible aging OA mice does not show OA phenotypes or common gait changes, but with some other gait differences.
A
TM-
TM+
200 µm
TM-
TM+
p = 0.34
B
C
Spatial
Temporal
**
p = 0.61
p = 0.0067
p = 0.53
p = 0.15
D
Others
***
**
p = 0.57
p = 0.68
p = 0.00039
p = 0.0030
E
Stride
Frequency
Stride time
Stride Length
COG
TM-
TM+
p = 0.41
p = 0.41
p = 0.37
p = 0.41
